# Autonomous Bioluminescent Expression of the Bacterial Luciferase Gene Cassette (*lux*) in a Mammalian Cell Line

**DOI:** 10.1371/journal.pone.0012441

**Published:** 2010-08-27

**Authors:** Dan M. Close, Stacey S. Patterson, Steven Ripp, Seung J. Baek, John Sanseverino, Gary S. Sayler

**Affiliations:** 1 The Center for Environmental Biotechnology, The University of Tennessee, Knoxville, Tennessee, United States of America; 2 Department of Pathobiology, The University of Tennessee College of Veterinary Medicine, Knoxville Tennessee, United States of America; East Carolina University, United States of America

## Abstract

**Background:**

The bacterial luciferase (*lux*) gene cassette consists of five genes (*luxCDABE*) whose protein products synergistically generate bioluminescent light signals exclusive of supplementary substrate additions or exogenous manipulations. Historically expressible only in prokaryotes, the *lux* operon was re-synthesized through a process of multi-bicistronic, codon-optimization to demonstrate for the first time self-directed bioluminescence emission in a mammalian HEK293 cell line *in vitro* and *in vivo*.

**Methodology/Principal Findings:**

Autonomous *in vitro* light production was shown to be 12-fold greater than the observable background associated with untransfected control cells. The availability of reduced riboflavin phosphate (FMNH_2_) was identified as the limiting bioluminescence substrate in the mammalian cell environment even after the addition of a constitutively expressed flavin reductase gene (*frp*) from *Vibrio harveyi*. FMNH_2_ supplementation led to a 151-fold increase in bioluminescence in cells expressing mammalian codon-optimized *luxCDE* and *frp* genes. When injected subcutaneously into nude mice, *in vivo* optical imaging permitted near instantaneous light detection that persisted independently for the 60 min length of the assay with negligible background.

**Conclusions/Significance:**

The speed, longevity, and self-sufficiency of *lux* expression in the mammalian cellular environment provides a viable and powerful alternative for real-time target visualization not currently offered by existing bioluminescent and fluorescent imaging technologies.

## Introduction

*In vivo* optical imaging is becoming increasingly utilized as a method for modern biomedical research. This process, which involves the non-invasive interrogation of animal subjects using light emitted either naturally from a luciferase protein or following excitation of a fluorescent protein or dye, has been applied to the study of a wide range of biological processes such as gene function, drug discovery and development, cellular trafficking, protein-protein interactions, and especially tumorigenesis and cancer treatment [1]. While the detection limits and resolution of charge coupled devices (CCDs) has increased greatly in recent years [2], there have been relatively few introductions of improved imaging compounds that function as light production centers within an animal subject *in vivo*.

Generally, the currently available imaging compounds can be divided into two classes: those containing luciferase proteins (capable of producing bioluminescent light without exogenous excitation) and those containing fluorescent compounds (dyes or proteins that require an initial excitation followed by emission at a given wavelength). For mammalian-based whole animal imaging, fluorescent compounds are limited due to high levels of background fluorescence from endogenous biological structures upon excitation [3]. Although dyes have been developed and employed that fluoresce in the near infrared wavelengths [4], [5] where light absorption is lowest in mammalian tissues, they can become increasingly diffuse during the process of cellular division, negating their usefulness in long term monitoring studies [1]. In contrast, luciferase proteins are highly amenable towards *in vivo* optical imaging (referred to as bioluminescent imaging or BLI) because they produce a controllable light signal in cells with little to no background bioluminescence, thus allowing for remarkably sensitive detection [6]. While historically the luciferase proteins used have been based on beetle luciferases (i.e., firefly or click beetle luciferase) or marine aequorin-like proteins (those that utilize coelenterazine), these each possess disadvantages when applied to whole animal BLI. For example, the popular firefly luciferase protein is heat labile when incubated under whole animal BLI imaging conditions, and can display a half life as short as 3 min in its native state at 37°C [7]. Coelenterazine-stimulated luciferases are similarly handicapped in regards to long-term monitoring, as it has been reported that their rapid uptake of coelenterazine necessitates prompt imaging following substrate injection [8]. Applications of both these luciferase systems also suffer from the drawback that they require addition of an exogenous substrate to produce a detectable light signal. This current work reports for the first time that a modified bacterial luciferase gene cassette can be expressed in mammalian cells in culture or in whole animal BLI without the use of exogenous substrates or coincident infection with a bacterial host, thus overcoming the limitations imposed by currently available luciferase-based BLI assays.

Setting the bacterial bioluminescence system apart from other bioluminescent systems such as firefly luciferase and aequorin is its ability to self-synthesize all of the substrates required for the production of light. While the luciferase component is a heterodimer formed from the products of the *luxA* and *luxB* genes, its only required substrates are molecular oxygen, reduced riboflavin phosphate (FMNH_2_), and a long chain aliphatic aldehyde. Oxygen and FMNH_2_ are naturally occurring products within the cell while the *luxCDE* gene products produce and regenerate the aldehyde substrate using endogenous aliphatic compounds initially targeted to lipid biogenesis. To produce light, the luciferase protein first binds FMNH_2_, followed by O_2_, and then the synthesized aldehyde. This allows the *lux* cassette to utilize only endogenous materials to form an intermediate complex that then slowly oxidizes to generate light at a wavelength of 490 nm as a byproduct [9]. The overall reaction can be summarized as:
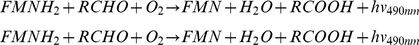



Realizing the distinct advantages bacterial luciferase would afford as a eukaryotic reporter, many groups have attempted to express the luciferase (*luxAB*) component of the *lux* system using either fusion proteins [10], [11], [12], [13] or multiple plasmids [14], [15], but with minimal success over the last twenty years. Although the use of *lux* in the study of bacterial infection of a mammalian host has been demonstrated using whole animal BLI [16], its functionality has not been demonstrated outside of a bacterial host until now. Recently, successful expression of a mammalian optimized luciferase dimer in an HEK293 cell line has provided for the limited use of *lux* as a mammalian bioluminescent reporter system, although the addition of luciferin in a manner similar to firefly luciferase is still required [17]. To fully exploit the advantages of bacterial luciferase, all five genes (*luxCDABE*) of the *lux* operon must be expressed simultaneously. Here it is demonstrated that codon-optimized, poly-bicistronic expression of the full *lux* cassette produces all of the products required for autonomous bioluminescent production in a mammalian background. We further demonstrate that cells expressing the full *lux* cassette can be applied towards whole animal BLI without the need for substrate addition, thus overcoming the limitations imposed by currently available luciferase-based whole animal BLI probes.

## Results

### Codon Optimization of the *luxCDE* and *frp* Genes

A major concern prior to mammalian expression was the thermostability of the luciferase proteins at the mammalian temperature optimum of 37°C. Traditionally, the marine dwelling bacterium *Vibrio harveyi* has been used as the cloning platform for *lux*-based manipulations, but its lack of protein thermostability above 30°C is not conducive for mammalian expression. Constructs were therefore derived from the genes of the terrestrial bacterium *Photorhabdus luminescens*, which, unlike *V. harveyi*, maintains *lux* protein product stability at 37°C [18]. While this species does not contain an associated NAD(P)H:Flavin reductase gene like *V. harveyi*, previous work suggested that incorporation of the *V. harveyi* oxidoreductase gene (*frp*) can increase bioluminescent output in lower eukaryotic systems [19]. Therefore, the *frp* gene was included in our constructs to shift the intracellular FMN/FMNH_2_ equilibrium to a more reduced state to provide an increased level of the required FMNH_2_ substrate.

Prior to expression, the *P. luminescens luxCDE* and *V. harveyi frp* gene sequences were interrogated for exogenic probability and the presence of interfering genetic regulatory signals upon expression in a mammalian cellular background using the GENSCAN algorithm (http://genes.mit.edu). Predictions indicated that the genes would not be efficiently expressed in a mammalian cell line in their wild-type form (Table 1). To promote improved transcriptional and translational expression, all genes were synthesized *de novo* using codon-optimized sequences (Figures S1, S2, S3, S4), as this process has previously been used to increase transcription and translation of a variety of native and non-native genes in mammalian cells [17], [20], [21], [22], [23]. Following optimization, GENSCAN analysis predicted a significantly higher translational level in the mammalian cellular background than the wild-type sequences, including the predicted full-length expression of the *luxE* gene due to the removal of regulatory signals (Table 1).

**Table 1:** Predicted expression of the of *lux* genes before and after codon-optimization.

Gene	Predicted Start Position	Length	% GC	Number of Nucleotide Substitutions	Probability of Recognition as an Exon	Exon Score
wt*luxC*	1	1443	37%	N/A	0.921	50.78
co*luxC*	1	1443	60%	449	0.999	360.72
wt*luxD*	1	924	38%	N/A	0.875	29.53
co*luxD*	1	924	59%	294	0.999	238.19
wt*luxE*	102	1087	38%	N/A	0.443	33.11
co*luxE*	1	1113	60%	331	0.999	271.01
wt*frp*	1	613	47%	N/A	0.715	30.70
co*frp*	1	723	64%	249	0.999	179.43

GENSCAN translation prediction scores for expression of wild-type (wt) and codon-optimized (co) *lux* genes in a mammalian host cell (http://genes.mit.edu). Predicted start position is the predicted nucleotide sequence location for initiation based on the transcription of the nucleotide gene sequence. The predicted truncation in length of the wild-type *luxE* gene was due to the presence of a non-mammalian preferred start codon in the original gene sequence. Exon score interpretation: 0–50 weak, 50–100 moderate, >100 strong. Additional length in the codon-optimized version of the *frp* gene is the result of additional nucleotides added to introduce new restriction enzyme sites for improved cloning efficiency. N/A - not applicable.

### Construction and Validation of Bicistronic Expression Vectors

**Figure 1:**
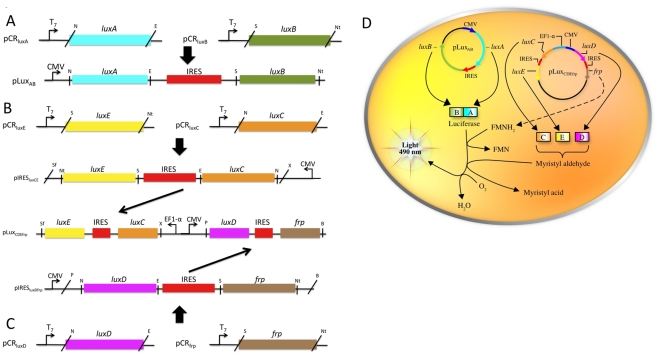
Schematic showing construction and expression of the full *lux* cassette using a two-plasmid system. The two-plasmid system takes advantage of IRES-based bicistronic expression to drive transcription/translation of all the genes required for autonomous bioluminescent production. (A) The pLux_AB_ plasmid contains the genes responsible for production of the luciferase protein. Individual *luxA* and *luxB* genes were removed from their respective vectors and ligated into the pIRES vector using the unique *Nhe*I (N) and *EcoR*I (E) or *Sal*I (S) and *Not*I (Nt) restriction sites. (B) The pLux_CDEfrp_ plasmid expresses the genes required for production and regeneration of the aldehyde and FMNH_2_ substrates. The individual *luxE* and *luxC* genes were cloned into a pIRES vector using the unique *Not*I (Nt) and *Sal*I (S) or *Nhe*I (N) and *EcoR*I (E) restriction sites. (C) A second pIRES vector was created that contained the *luxD* and *frp* genes inserted at the same sites. The entire *luxC*-IRES-*luxE* fragment was then inserted under the control of the EF1-α promoter in pBudCE4.1 using the unique *Xho*I (X) and *Sfi*I (Sf) restriction sites, while the *luxD*-IRES-*frp* fragment was inserted under the control of the CMV promoter using the unique *Pst*I (P) and *BamH*I (B) restriction sites. (D) When expressed simultaneously, these two plasmids produce all the proteins required for bioluminescence expression by utilizing the FMN and molecular oxygen stores supplied endogenously by the host cell.

Following codon-optimization, the *lux* genes were grouped into pairs and separated by internal ribosomal entry site (IRES) elements from the encephalomyocarditis virus to more efficiently drive translation in the mammalian cell environment (Figure 1A–C). This expression strategy was chosen because it has previously been demonstrated that the use of intervening IRES elements allowed bicistronic expression of the *lux* genes in the lower eukaryote *Saccharomyces cerevisiae* [19]. The presence of intervening IRES elements allows for transcription of a fused mRNA product from a single promoter, followed by cap-independent translation of the gene distal to the promoter concurrent with traditional cap-dependent expression of the promoter proximal gene [24]. To avoid problems associated with transfection efficiency and stable maintenance of a single large plasmid, the *lux* cassette was divided between two plasmids prior to transfection. This strategy also provided for the translation of proteins requiring proximity for function to occur as near to one another as could be controlled. The first plasmid, pLux_CDEfrp_:CO (Figure S5A), contained the codon-optimized *luxCDEfrp* genes responsible for production and regeneration of the required aldehyde substrate and increased turnover of the FMNH_2_ substrate. The *luxD* gene encodes a transferase responsible for attachment of an endogenous myristyl group to water to form myristyl acid. The *luxE* gene encodes a synthase that then activates the acid via addition of an AMP group in order to prepare the reduction of the activated acid to aldehyde through the reductase encoded by *luxC*. The *frp* gene operates separately to cycle FMN to FMNH_2_ in the cytosol [9]. To validate the GENSCAN predictions and establish the effectiveness of the codon-optimization process, an alternate version of this plasmid was created, pLux_CDEfrp_:WT (Figure S5B), which uses the wild-type *P. luminescens* and *V. harveyi* gene sequences. A second plasmid, pLux_AB_, contained the *luxA* and *luxB* genes separated by an IRES element for bicistronic expression of the luciferase dimer subunits responsible for providing the scaffolding and enzymatically eliciting the production of light following the binding of all required substrates. When expressed *in vivo*, pLux_AB_ in combination with pLux_CDEfrp_:CO or pLux_CDEfrp_:WT contains all of the genes required for autonomous bioluminescent expression (Figure 1D). HEK293 cells were subsequently co-transfected with the pLux_CDEfrp_:CO/pLux_AB_ or pLux_CDEfrp_:WT/pLux_AB_ plasmid combinations and selected by antibiotic resistance. A lineage of HEK293 cells remained untransfected for use as a control to determine background in the presence of standardized amounts of cellular material.

To determine and compare the bioluminescent output kinetics of HEK293 cells containing the *luxCDEfrp* genes in either their wild-type (pLux_CDEfrp_:WT) or codon-optimized (pLux_CDEfrp_:CO) form, cells were propagated under identical conditions, harvested, and resuspended directly in a cuvette for measurement of bioluminescence against a standard photomultiplier tube interface. Cells containing pLux_CDEfrp_:CO/pLux_AB_ showed an average bioluminescent production 12-fold greater than background in the presence of untransfected control cells and 9-fold greater than the bioluminescent production of their wild-type counterparts (Table 2). The superior bioluminescent production by cells containing pLux_CDEfrp_:CO/pLux_AB_ validates our dual plasmid, bicistronic, codon-optimized expression strategy and substantiates our hypothesis that the full bacterial *lux* cassette can be designed for functional autonomous expression in a mammalian cell line.

**Table 2:** Bioluminescent production from unsupplemented HEK293 cells expressing *P. luminescens lux* genes.

Cell Line	Bioluminescent Detection (RLU/sec)
Cell Free Media	745 (±63)
Untransfected HEK293 Cells	655 (±44)
HEK293+pLux_AB_+pLux_CDEfrp_:WT	884 (±44)
HEK293+pLux_AB_+pLux_CDEfrp_:CO	7600 (±1241)

Actively growing HEK293 cells expressing various combinations of *P. luminescens lux* genes were harvested from culture and directly assayed for *in vivo* bioluminescent production. Lower levels of bioluminescent output from untransfected cells as opposed to cell free medium are most likely the result of background light absorption from endogenous chromophoric material in the cells. All values are the average of at least three trials and are reported with the standard error of the mean.

### Growth Curve Analysis of *lux*-Containing HEK293 Cells

To determine if the maintenance and expression of full complements of *lux* genes was detrimental to cellular growth rates in HEK293 cells, the rates of growth among wild-type, pLux_CDEfrp_:CO, and pLux_CDEfrp_:WT containing cells was monitored over the course of a normal passage cycle. It was hypothesized that any adverse effects from production of aldehyde or increased presence of FMNH_2_ resulting from the expression of the pLux_CDEfrp_ plasmid would result in a slowed growth rate relative to the wild-type HEK293 cell line. No significant difference in the rates of growth was observed among any of the cell lines tested (Figure S6), suggesting that any adverse effects resulting from expression of the *luxCDEfrp* genes are minimal in regards to cellular growth and metabolism.

### *in vitro* Bioluminescent Imaging

**Figure 2:**
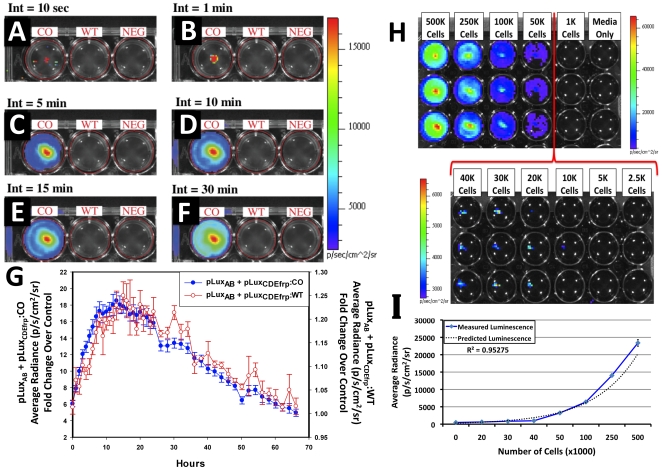
*in vitro* bioluminescent imaging of *lux* cassette containing cells. pLux_CDEfrp_:CO/pLux_AB_ containing (CO), pLux_CDEfrp_:WT/pLux_AB_ containing (WT), and untransfected negative control (NEG) HEK293 cells were plated in 24-well tissue culture plates and integrated for (A) 10 sec, (B) 1 min, (C) 5 min, (D) 10 min, (E) 15 min, and (F) 30 min. Bioluminescence from cells co-transfected with pLux_CDEfrp_:CO/pLux_AB_ was distinguishable from background in the presence of untransfected cells after 10 sec and showed no increase in background detection even after a 30 min integration time. Long term *in vitro* expression (G) demonstrates the temporal longevity of the signal without exogenous amendment. The minimum detectable number of bioluminescent cells was determined (H) by plating a range of cell concentrations in equal volumes of media in triplicate (downward columns) in an opaque 24-well tissue culture plate. The minimum number of cells that could be consistently detected was approximately 20,000. Average radiance was shown to correlate with plated cell numbers (I), yielding an R^2^ value of 0.95275.

For a *lux*-based system to function as a reporter in whole animal BLI, the resulting signal must be detectable using commercially available equipment designed for this purpose and be easily distinguishable from background light emissions. To determine if this was the case in HEK293 cells expressing full *lux* cassettes, approximately equal numbers of cells containing either codon-optimized or wild-type *lux* genes were plated in 24-well tissue culture plates and compared with untransfected cells as a negative control for background. The bioluminescent signal from cells co-transfected with codon-optimized *luxCDEfrp* was differentially detectable from background using a 10 sec integration time (Figure 2A) and increased in magnitude with no appreciable increase in background up to integration times of 30 min (Figure 2B–F). To determine the maximal duration of the bioluminescent signal during constitutive expression under experimental conditions, approximately equal numbers of HEK293 cells in either their untransfected state or containing pLux_AB_ co-transfected with either pLux_CDEfrp_:WT or pLux_CDEfrp_:CO were continually monitored for bioluminescence production (Figure 2G) in an IVIS Lumina imaging system using a stage temperature of 37°C to mimic as closely as possible their normal growth conditions. Cells containing the *lux* cassette genes demonstrated bioluminescent output over an approximate three-day period without any exogenous input. Peak bioluminescent output was achieved between 12 and 13 h for both the codon-optimized and wild-type containing cell lines, however, following peak bioluminescent output a slow decrease in bioluminescent production over time was observed. This decrease is presumably due to a combination of the inability to reliably regulate the air temperature, CO_2_ levels, and humidity in the imaging system, and the continued depletion of nutrients from the media during the normal process of cellular growth and metabolism. While the bioluminescent output of cells containing pLux_CDEfrp_:WT/pLux_AB_ was of a lesser magnitude than that of their codon-optimized counterparts over this time period, their bioluminescent expression profiles were similar under the same conditions, suggesting that the codon-optimization process had not significantly altered the function of the *lux* proteins *in vivo*.

### Determination of Minimal Detectable Cell Number *in vitro*

To be useful as an optical reporter, cells expressing bioluminescence must be detectable over a dynamic population range. To determine the minimum detectable cell number, HEK293 cells containing pLux_CDEfrp_:CO/pLux_AB_ at concentrations ranging from 1,000 to 500,000 cells were plated in triplicate in equal volumes of media over a constant surface area and imaged over a 10 min integration time. The minimum number of cells reliably detected above background was approximately 20,000 although some visible signal was detected at approximately 10,000 cells in at least one case (Figure 2H).

### Correlation of Bioluminescent Flux to Cell Number

A major advantage imparted by the use of bioluminescent or fluorescent-tagged reporter cells is that they allow an investigator to approximately quantify the population size of those cells noninvasively in a living host. For this approximation to be made using a *lux*-based system, it must be demonstrated that the bioluminescent flux of the cell population correlates tightly with the overall population size. To determine if this is the case in HEK293 cells constitutively expressing codon-optimized bacterial luciferase genes, the average radiance of cells producing a visibly detectable bioluminescent signal was determined over cell concentrations ranging from 500,000 to 1,000 cells. The average radiance closely correlated with the number of cells present (R^2^ = 0.95275) over all visibly detectable cell numbers tested (Figure 2I).

### Whole Animal Bioluminescent Imaging

**Figure 3:**
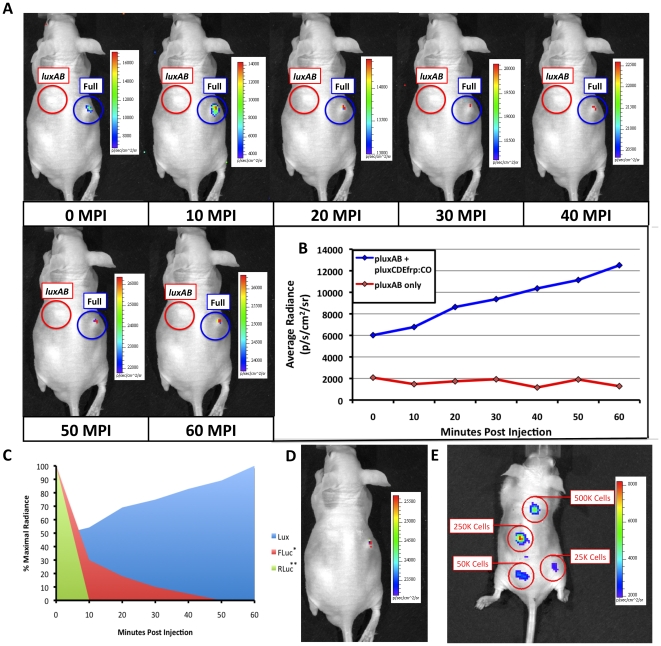
*in vivo* bioluminescent imaging using HEK293 cell expression of mammalian-adapted *lux*. (A) HEK293 cells containing the mammalian adapted pLux_CDEfrp_:CO/pLux_AB_ cassette (Full) were subcutaneously injected into nude mice and imaged. Detection occurred nearly immediately (<10 sec) post-injection and remained visible up to the 60 min time point of the imaging assay. HEK293 cells containing only the pLux_AB_ plasmid (*luxAB*) were subcutaneously injected into the same mouse as a negative control. Note that the automatic scaling of signal intensity differs among images, therefore creating the false appearance that image intensity is decreasing after the 10 min post-injection time point when in fact it continually increases as shown in panel (B). (C) Comparison of mammalian-adapted *lux*-based bioluminescence from HEK293 cells versus published data on the expression of FLuc (*[25]) and RLuc (**[8]) tagged cells over the 60 min course of the assay. (D) Upon termination of the assay 60 min post injection, the bioluminescent signal from HEK293 cells expressing the full complement of *lux* genes was detectable using an integration time as low as 30 sec. (E) Subcutaneous injection of HEK293 cells containing pLux_CDEfrp_:CO/pLux_AB_ at concentrations ranging from 500,000 to 25,000 cells in 100 µl volumes of PBS demonstrated a tested lower limit of detection of 25,000 cells using a 10 min integration time. MPI, minutes post injection.

Although *lux* has been previously used in whole animal BLI [16], this is the first demonstration of its functionality outside of a bacterial host. Bacteria-free expression of this genetic system assures that the results seen are directly related to the object of study, and are not artifacts of a host-pathogen interaction stemming from the previously required bacterial infection. To demonstrate this functionality, 5 week old nude mice were subcutaneously injected with HEK293 cells co-transfected with pLux_CDEfrp_:CO/pLux_AB_ or pLux_AB_ alone and imaged. Cells containing only pLux_AB_ were injected as a negative control to determine if the substrates supplied by the *luxCDEfrp* genes in the pLux_CDEfrp_ plasmid were capable of being scavenged from endogenously available stocks within the host in the presence of the luciferase dimer formed by the products of the *luxAB* genes on the pLux_AB_ plasmid. Bioluminescent signal emission from injected pLux_CDEfrp_:CO/pLux_AB_ HEK293 cell lines was detectable immediately (<10 sec) following injection (Figure 3A), mirroring the results of subcutaneous tumor mimic bioluminescence from firefly luciferase (FLuc)-tagged [25] and Renilla luciferase (RLuc)-tagged [8] cells following intravenous (IV) injection of their D-luciferin or coelenterazine substrates, respectfully. Following injection, the *lux* signal increased slowly in intensity over the full 60 min course of the assay (Figure 3B). This is in contrast to FLuc-based bioluminescent signals that exhibit a steady decline over the same period following IV injection of D-luciferin to a level ∼20% of their initial intensity [25]. RLuc bioluminescence is even more temporally limited and subsides within 5 min following IV injection of coelenterazine [8] (Figure 3C). In contrast, the *lux* bioluminescent signal remained detectable 60 min after injection using integration times as low as 30 sec (Figure 3D). Conversely, FLuc signals are asymptotically approaching their minimum [25] and RLuc signals have become fully attenuated [8] by 30 min, thus making imaging at all but the shortest post-injection incubation times impossible (Figure 3C). It is important to note that the duration of the bioluminescent signal in FLuc containing systems can be extended by using a subcutaneous or intraperitoneal injection of luciferin, however, each injection route also produces a different bioluminescent emission profile over time [25]. *lux*-based systems are not subject to these effects because they forgo the addition of exogenous substrates to trigger bioluminescence. The lack of a signal after injection of cells expressing only pLux_AB_ at any of the time points sampled (Figure 3A) confirms that the luciferase dimer alone is not capable of producing unintended bioluminescence above the background levels of light detection by scavenging endogenously available substrates. These results demonstrate the utility of the *lux* system in providing bioluminescent data on relatively prolonged time scales without the potentially error-inducing requirement of disturbing the experimental environment to invasively inject additional luciferin substrate.

### Determination of Minimal Detectable Cell Number *in vivo*

Having illustrated the ability to reliably detect at least 20,000 cells in a tissue culture setting (Figure 2H), the minimum detectable number of cells in small animal models remained to be determined. The detection of bioluminescent cells following subcutaneous injection is more difficult than detection in a culture setting due to the increased presence of chromophoric material leading to higher absorption of emitted photons as they must travel through more tissue to reach the detector. Subcutaneous injections of decreasing numbers of cells into a nude mouse model revealed that the introduction of at least 25,000 cells was capable of producing a detectable signal (Figure 3E). As predicted from the correlation of cell number to bioluminescent flux, injection of higher cell concentrations produced larger bioluminescent signals over identical integration times.

### Oxidoreductase Supplemented *in vitro* Light Assays

**Figure 4:**
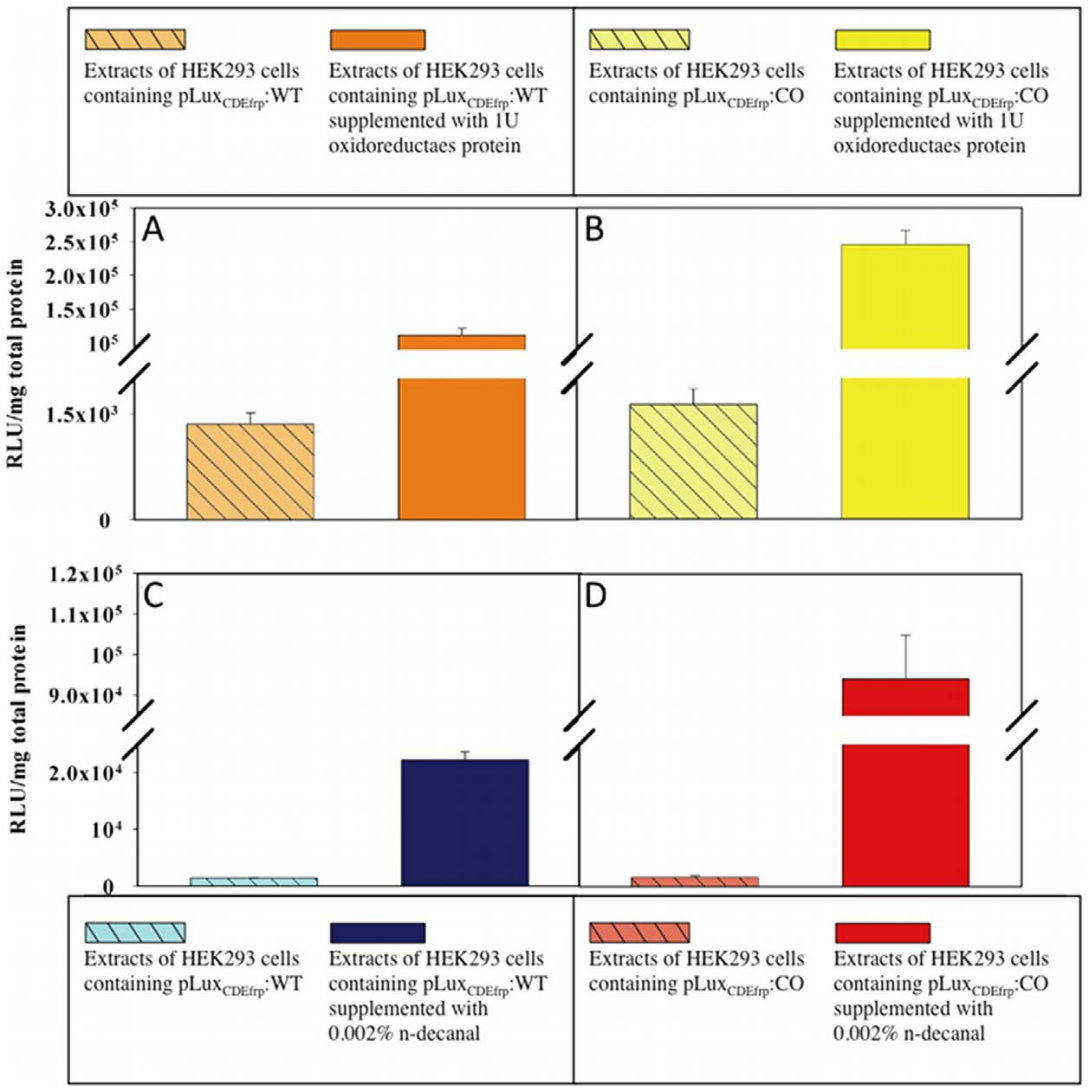
Supplementation assays demonstrating the functionality of the *luxCDEfrp* genes in the mammalian cell environment. Supplementation with 1 U oxidoreductase protein significantly increased light output in cell extracts from (A) wild-type and (B) codon-optimized cell lines. Supplementation with 0.002% n-decanal resulted in increased bioluminescent output in both the (C) wild-type and (D) codon-optimized cell extracts as well, but at a lower magnitude than oxidoreductase supplementation. Values are the average of four trials, and are reported with the standard error of the mean.

Previous work with the *lux* system in lower eukaryotes has shown the initial substrate, FMNH_2_, to be a limiting reagent in the reaction [19]. To determine if this was the case in HEK293 cells, *in vitro* supplementation assays were performed with the addition of 1 U of NAD(P)H:Flavin oxidoreductase protein isolated from *Photobacterium fischeri*. Protein extracts from cells containing the *lux* genes in either their codon-optimized or wild-type forms were subjected to *in vitro* analysis to determine if the addition of oxidoreductase protein could improve light output. Upon addition of the flavin oxidoreductase protein, the average bioluminescent output increased from 1,400 (±200) RLU/mg total protein to 111,500 (±10,500) RLU/mg total protein in pLux_CDEfrp_:WT containing cells (Figure 4A) and from 1,600 (±200) RLU/mg total protein to 245,000 (±20,500) RLU/mg total protein in pLux_CDEfrp_:CO containing cells (Figure 4B).

### Aldehyde Supplemented *in vitro* Light Assays

The synthesized co-substrate required for light production in the *lux* system is a long chain aliphatic aldehyde that binds to the luciferase and is oxidized [9]. The ability, conferred by the *luxCDE* genes, to produce and recycle the aldehyde substrate endogenously makes *lux* a uniquely beneficial reporter system. To assay for the production of aldehyde, cell extracts were supplemented with 0.002% (w/v) n-decanal, as this has previously been shown capable of functioning in place of the natural aldehyde substrate [17], [19], [26], [27], [28]. When supplied with aldehyde, both the pLux_CDEfrp_:WT and pLux_CDEfrp_:CO containing cell extracts showed increases in bioluminescent output. Cell extracts from wild-type containing cells showed an increase from 1,400 (±200) RLU/mg total protein to 22,000 (±1,500) RLU/mg total protein (Figure 4C). Extracts from codon-optimized cells increased from the baseline of 1,600 (±200) RLU/mg total protein to 94,000 (±10,800) RLU/mg total protein (Figure 4D).

## Discussion

Development of the *lux* cassette into a functional and autonomous mammalian bioluminescent system provides researchers a unique new tool that allows for real-time monitoring of bioluminescence from whole animals or cell cultures without exogenous substrate addition or cell lysis. The first step in the creation of this reporter was the functional demonstration of the luciferase heterodimer formed by the *luxAB* genes [17]. This set the stage for the use of *lux* in eukaryotic cells as a non-autonomous reporter system via the addition of aldehyde. Since that time, the production of aldehyde has been demonstrated in *S. cerevisiae* [19], leading to the development of the first eukaryotic *lux*-based autonomous reporter system. Here we demonstrate for the first time that expression of codon-optimized forms of the *luxCDE* genes from *P. luminescens* and the *frp* gene from *V. harveyi* are capable of producing sufficient levels of the aldehyde and FMNH_2_ substrates required to drive light production autonomously in mammalian cells. We further demonstrate that these bioluminescent cells can be applied in whole animal BLI without the need for substrate addition.

While the addition of *luxCDEfrp* to cells containing *luxAB* demonstrates light emission at a level 12-fold greater than background (Table 2), it is clear that concentrations above the available levels of either the FMNH_2_ (Figure 4A and C) or aldehyde substrates (Figure 4B and D) will result in increased bioluminescent output. However, an increase in aldehyde production can be cytotoxic, as has been demonstrated in *luxAB* containing *S. cerevisiae* and *Caenorhabditis elegans* cells [29]. This may lead to a scenario where the *luxCDE* containing cells that most efficiently produce the aldehyde substrate are selected against during the initial period of growth following transfection with *luxCDEfrp* due to slowed growth and/or elevated cytotoxicity. The increased presence of aldehyde may therefore cause those cells capable of most efficiently producing aldehyde to inhibit their own growth, mimicking the effects of antibiotic selection and causing them to be out-competed in culture by cells expressing lower levels of aldehyde production. Mathematical models of the *lux* system have indicated that the production of light is much more sensitive to the aldehyde turnover rates modulated by the *luxCE* genes responsible for encoding the reductase and synthase that convert the myristyl acid to a myristyl aldehyde than it is to the concentration of luciferase dimer formed by the *luxAB* genes responsible for catalyzing the reaction and facilitating the production of light. The model predicts that a reduction in the concentration of the *luxC* or *luxE* gene products will lead to a drastic reduction in light output [30]. If true, then it is hypothesized that the cytotoxicity of aldehyde within the cell may be a non-issue in regards to selecting cell lines that can function in bioluminescent imaging assays. Cells with cytotoxic levels of aldehyde production will be removed early in the selection process due to slow growth rates and inability to compete with faster growing cell lines during the antibiotic selection phase following transfection. Similarly, cells with low levels of *luxCDE* expression will not generate high levels of bioluminescence during *in vitro* screening of *luxCDEfrp* containing cell lines. This would tend to encourage only the selection of cell lineages capable of producing just enough aldehyde to drive the *lux* reaction, but not enough to impair cellular growth and function, as platforms for biosensor development. Experiments aimed at determining if expression of the *lux* cassette genes (and, by extension, the products of their associated reactions) altered cellular metabolism and growth rates have supported these predictions. This investigation revealed no significant variation among the growth rates of untransfected HEK293 cells or those expressing either pLux_CDEfrp_:WT/pLux_AB_ or pLux_CDEfrp_:CO/pLux_AB_ at levels capable of supporting continuous bioluminescent production (Figure S6). These cells are necessarily producing the required aldehyde substrate as demonstrated by their constitutive bioluminescent production, but do not show a detectable difference in their rate of growth when compared to cells that are grown under identical conditions but without the *luxCDE* genes required for the production and maintenance of the aldehyde substrate.

As shown in Figure 4, the availability of FMNH_2_ appears to contribute as a limiting reagent for the *lux* reaction in a mammalian cell environment. Supplementation with as little as 1 U of oxidoreductase protein *in vitro* led to relatively large (up to 151-fold) increases in bioluminescent output levels, while supplementation with 0.002% n-decanal produced less substantial (up to 58-fold) increases in light production. When supplemented with additional oxidoreductase protein to drive the turnover of FMN to FMNH_2_, the average production of light increased by 82-fold in wild-type cell extracts (Figure 4A) and by 151-fold in extracts from cells containing codon-optimized *lux* genes (Figure 4B). The increases in light production attributed to additional FMNH_2_ were consistently of greater magnitude than those associated with aldehyde supplementation. The highest increase in light output achieved through addition of n-decanal was 58-fold in cells containing codon-optimized genes (Figure 4D), compared with only a 16-fold increase in light output from cell extracts co-transfected with the wild-type genes (Figure 4C). These results suggest that codon optimization of the remaining *luxCDE* genes from *P. luminescens* allows for more efficient processing of the available substrates in the mammalian cell environment, but does not allow for production levels that rival the ideal conditions of *in vitro* substrate supplementation where the bioluminescent output would be limited only by the efficiency of the LuxAB luciferase dimer. When supplemented with identical levels of aldehyde, cell extracts containing codon-optimized *luxCDEfrp* genes were able to produce over four times as much light as those containing the wild-type genes (Figure 4C and D). A similar result was obtained under oxidoreductase supplementation, with extracts from the codon-optimized cell lines producing over twice as much light as their wild-type counterparts (Figure 4A and B).

When codon-optimized *lux* containing HEK293 cells were used in cell culture, concentrations of approximately 20,000 cells were reliably detected in 1 ml of media immediately using a 10 min integration time (Figure 2H). Increasing cell numbers in the same volume and area correlated with measured levels of bioluminescence emission, allowing one to predict the total cell number in a given sample from the measured average radiance (Figure 2I) and permitting non-invasive estimation of target size based on bioluminescent measurements.

When the same bioluminescent cell lines were applied in whole animal BLI, the low levels of detectable background signal and deficit of endogenous bioluminescent production associated with mammalian cells enabled *lux*-based bioluminescence to remain detectable despite relatively low levels of aldehyde and FMNH_2_ substrate availability as compared to ideal, *in vitro* supplemented conditions. This sensitivity was demonstrated both in cell culture and under subcutaneous whole animal BLI conditions where very little light is produced due to attenuation of the bioluminescent signal by absorption from endogenous chromophores [31]. We have demonstrated here that cells co-transfected with the codon-optimized *luxCDEfrp* genes can produce a lasting signal that can be amplified over integration times as long as 30 min with little to no background to interfere with signal acquisition (Figure 2F) in a cell culture setting. However, it is important to note that the bioluminescent signal from this reaction is produced at 490 nm. This is relatively blue-shifted as compared to the Luc-based bioluminescent probes that display their peak luminescent signal at 560 nm. The shorter wavelength of the *lux*-based signal has a greater chance of becoming attenuated within the tissue and therefore may not be as easily detected if it is used in deeper tissue applications (such as intraperitoneal or intraorganeller injections), and may require longer integration times to achieve the same level of detection as a longer wavelength reporter would when injected subcutaneously. For instance, it has been reported that a single cell expressing FLuc can be detected following subcutaneous injection [32], whereas we have demonstrated that the approximate lower level of detection for *lux*-tagged cells is closer to 25,000 cells, most likely due to the lower quantum efficiency of the *lux* bioluminescent system coupled with the higher rates of attenuation due to absorption at the emission wavelength of 490 nm. Therefore, the goals of a particular experiment should be carefully weighed before applying a *lux*-based bioluminescent reporter. While a *lux*-based system can produce a continuous bioluminescent signal over prolonged time periods without being subjected to the dynamic effects of repeated luciferin injections, it may not be appropriate for situations with high levels of signal attenuation due to its lower emission wavelength.

Despite such drawbacks, the use of cells expressing bacterial luciferase genes as a probe for whole animal BLI solves many of the problems associated with the currently available luciferase-based imaging systems. Previous work with *lux* genes isolated from *P. luminescens* has demonstrated that the luciferase is thermostable at the 37°C temperature required for mammalian imaging experiments [33]. This prevents the associated loss of signal associated with the short half-life of the firefly luciferase, which has been shown to be thermolabile at 37°C in its native state [7]. In addition, the autonomous nature of bioluminescent production associated with the *lux* system circumvents continuous re-injection of the test animal with an exogenous luciferin substrate. This simultaneously reduces the amount of invasive injections required for imaging experiments, eliminates the detection of artificial results stemming from any non-specific biological reactions with the luciferin compound being administered, and negates the inability to compare otherwise similar experiments due to differential bioluminescent production kinetics based on dissimilar routes of substrate injection. Thus, the bacterial luciferase offers a more specific, longer lasting, and more humane luciferase-based reporter system than the currently available alternatives.

While mammalian-adapted bacterial luciferase gene expression has some notable disadvantages such as requisite introduction of multiple gene sequences and bioluminescent production at a wavelength that is relatively highly absorbed in mammalian tissues, it remains easily detectable using currently available imaging technology and offers several important advantages over the currently available reporter systems for prolonged expression without the cost or disturbance to the system associated with substrate administration. We have shown here that expression of the *luxCDE* genes in mammalian cells can produce a long chain aliphatic aldehyde that is available for use as a substrate for bioluminescent production and that incorporation of the *frp* gene increases this output. Codon optimization of these genes improves their performance and leads to an overall increase in light production as compared to their wild-type counterparts. When co-expressed with the *luxAB* genes responsible for formation of the luciferase heterodimer, aldehyde production occurs at a level capable of inducing autonomous light production, but not of high enough concentration to be adversely cytotoxic. When cells containing full complements of *lux* genes are enlisted as probes in whole animal BLI, they are easily detectable when introduced at levels comparable to cells expressing other currently employed target luciferase genes and allow for facile differentiation from background over prolonged integration times at 37°C, making them ideal reporter systems for cell culture, subcutaneous, or other low absorption environments that require prolonged, real-time monitoring without disruption.

## Materials and Methods

### Ethics statement

All animal work was performed in adherence to the institutional guidelines put forth by the animal care and use committee of the University of Tennessee. All animal research procedures were approved by the University of Tennessee Animal Care and Use Committee (protocol number 1411) and were in accordance with National Institutes of Health guidelines.

### Strain maintenance and growth

*Escherichia coli* cells were routinely grown in Luria Bertani (LB) broth with continuous shaking (200 rpm) at 37°C. When required, kanamycin or ampicillin was used at final concentrations of 40 and 100 µg/ml, respectfully, for selection of plasmid containing cells. Mammalian cell lines were propagated in Eagle's modified essential medium (EMEM) supplemented with 10% fetal bovine serum, 0.01 mM non-essential amino acids, and 0.01 mM sodium pyruvate. Cell growth was carried out at 37°C in a 5% CO_2_ environment and cells were passaged every 3–4 d upon reaching 80% confluence. Neomycin and/or zeocin were used for selection of transfected cells at concentrations of 500 µg/ml and 200 µg/ml, respectfully, as determined by kill curve analysis, for each antibiotic.

### Codon optimization of *lux* genes

Codon usage patterns in the *luxCDE* genes for *P. luminescens* and the flavin reductase gene (*frp*) from *V. harveyi* were compared to the highest 10% of expressed genes as represented in GenBank. Silent mutations at the DNA level that would alter native codon usage were plotted to more closely mimic the preferred mammalian codons while maintaining 100% amino acid identity with the bacterial protein sequences. When multiple codons were preferred in equal or near equal frequencies by mammalian genes, the codon for the optimized sequence was randomly selected from the available options. These optimized sequences were submitted and synthesized *de novo* by GeneArt and returned as synthetic DNA constructs inserted into unique *Kpn*I and *Sac*I restriction sites in pPCR-Script vectors (GeneArt). Codon-optimized versions of each gene were compared to their wild-type counterpart for predicted translational efficiency using the freely available GENSCAN software at http://genes.mit.edu. All sequences were deposited to GenBank under the following accession numbers GQ850533 (codon-optimized *luxC*), GQ850534 (codon-optimized *luxD*), GQ850535 (codon-optimized *luxE*), and GQ850536 (codon-optimized *frp*).

### Vector construction

Previously described [17] *P. luminescens luxA* and *luxB* genes partially codon-optimized for expression in human cell lines were obtained as a bicistronic operon in a pIRES vector (Clontech) and designated pLux_AB_. This vector includes an internal ribosomal entry site (IRES) for increased translation of downstream gene inserts. The remaining *P. luminescens* genes (*luxC*, *luxD*, *luxE*) and the flavin reductase gene (*frp*) were used in either their wild-type (wt) or codon optimized (co) states. co*luxC* was cloned into multiple cloning site (MCS) A of the pIRES vector using the unique *Nhe*I and *EcoR*I restriction sites (Figure 1A–C). co*luxE* was then inserted into MCS B using the unique *Sal*I and *Not*I restriction sites. This entire co*luxC*-IRES-co*luxE* sequence was then removed and ligated into pBudCE4.1 (Invitrogen) behind the human elongation factor 1α (EF-1α) promoter using unique *Xho*I and *Sfi*I restriction sites. A second pIRES vector was constructed by adding the co*luxD* gene to MCS A via the unique *Nhe*I and *EcoR*I restriction sites and the addition of co*frp* to MCS B using the unique *Sal*I and *Not*I restriction sites. This entire co*luxD*-IRES-co*frp* sequence was then inserted behind the pBudCE4.1 human cytomegalovirus immediate early promoter (CMV) using the unique *Pst*I and *BamH*I restriction sites to create pLux_CDEfrp_:CO. This process was repeated using wild-type codon usage versions of each of the genes to generate an identically oriented, but non-codon-optimized, vector referred to as pLux_CDEfrp_:WT.

### Mammalian cell transfection and selection of cell lines

Transfection was carried out in six-well Falcon tissue culture plates (Thermo-Fisher). HEK293 cells stably expressing the pLux_AB_ vector were passaged into each well at a concentration of approximately 1×10^5^ cells/well and grown to 90–95% confluence in complete medium as described above. pLux_CDEfrp_:CO and pLux_CDEfrp_:WT plasmid vectors were purified from 100 ml overnight cultures of *E. coli* using the Wizard Purefection plasmid purification system (Promega). On the day of transfection, cell medium was removed and replaced and vector DNA was introduced using Lipofectamine 2000 (Invitrogen). Twenty-four h post-transfection, the medium was removed and replaced with complete medium supplemented with the appropriate antibiotic. Selection of successfully transfected clones was performed by refreshing selective medium every 4–5 d until all untransfected cells had died. At this time, colonies of transfected cells were removed by scraping, transferred to individual 25 cm^2^ cell culture flasks, and grown in complete medium supplemented with the appropriate antibiotics.

### Growth curve analysis

Cells were harvested during exponential growth from a 75 cm^2^ tissue culture flask and split into four 25 cm^2^ tissue culture flasks at ∼5×10^4^ cells/cm^2^. At 24 h intervals, the cells were detached from the flasks by mechanical agitation and resuspended in 3 ml phosphate buffered saline (PBS). A 15 µl aliquot was removed and diluted into an equal volume of trypan blue. Cells were counted using a hemocytometer and the average of 4 counts was used to determine the total viable cell number.

### Protein extraction

Total protein was extracted from co-transfected pLux_CDEfrp_:CO/pLux_AB_ and pLux_CDEfrp_:WT/pLux_AB_ cell lines using a freeze/thaw procedure. Cells were first grown to confluence in 75 cm^2^ tissue culture flasks, then mechanically detached and resuspended in 10 ml of PBS. Following collection, cells were washed twice in 10 ml volumes of PBS, pelleted and resuspended in 1 ml PBS. These 1 ml aliquots of cells were subjected to three rounds of freezing in liquid nitrogen for 30 sec, followed by thawing in a 37°C water bath for 3 min. The resulting cell debris was pelleted by centrifugation at 14,000g for 10 min and the supernatant containing the soluble protein fraction was retained for analysis.

### Bioluminescence assays

Bioluminescence was measured using an FB14 luminometer (Zylux) with a 1 sec integration time. To prepare the sample for *in vitro* bioluminescent measurement, 400 µl of the isolated protein extract was combined with 500 µl of either oxidoreductase supplemented light assay solution containing 0.1 mM NAD(P)H, 4 µM FMN, 0.2% (w/v) BSA and 1 U of oxidoreductase protein isolated from *V. fischeri* (Roche), or oxidoreductase deficient light assay solution (distilled water substituted for the 1 U of oxidoreductase protein). Following the initial bioluminescent reading, samples were amended with 0.002% (w/v) n-decanal and the readings were continued to determine if additional aldehyde could increase light output. All bioluminescent signals were normalized to total protein concentration as determined by BCA protein assay (Pierce) and reported as relative light units (RLU)/mg total protein. All sample runs included processing of cell extracts from HEK293 cells stably transfected with pLux_AB_ as a control for light expression upon amendment. To prepare cells for *in vivo* bioluminescent measurement, the total cell contents of a 75 cm^2^ tissue culture flask were resuspended in 1 ml of Dulbecco's Modified Eagle Medium (DMEM) without phenol red supplemented with 10% fetal bovine serum, 0.01 mM non-essential amino acids, and 0.01 mM sodium pyruvate. A 15 µl aliquot of cells was removed and counted using a hemocytometer to allow all values to be normalized to viable cell counts. The remainder was used directly for bioluminescent measurement using the FB14 luminometer with a 1 sec integration time.

### Cell culture bioluminescent imaging

Photon counts were recorded using an IVIS Lumina *in vivo* imaging system and analyzed with Living Image 3.0 software (Caliper Life Sciences). Actively growing HEK293 cells expressing either pLux_CDEfrp_:CO/pLux_AB_, pLux_CDEfrp_:WT/pLux_AB_, or no exogenous DNA (untransfected, negative control) were trypsinized and harvested from 75 cm^2^ tissue culture flasks and approximately one million cells were plated in each of three wells in a 24-well tissue culture plate in DMEM without phenol red and supplemented with 10% fetal bovine serum, 0.01 mM non-essential amino acids, and 0.01 mM sodium pyruvate. Average radiance in photons/sec/cm^2^/sr was determined over 10 min intervals every hour for the first 24 h and every 2 h thereafter until radiance returned to the initial level to obtain representative counts. Untransfected HEK293 cells were included in all trials as negative controls to assay for background noise.

### Determination of minimal detectable cell number and correlation of bioluminescent flux to cell number *in vitro*

Actively growing HEK293 cells expressing pLux_CDEfrp_:CO/pLux_AB_ were trypsinized and harvested from 75 cm^2^ tissue culture flasks and counted using a hemocytometer. Using a 24-well tissue culture plate, groups of approximately either 500,000, 250,000, 100,000, 50,000, 40,000, 30,000, 20,000, 10,000, 5,000, 2,000, or 1,000 cells were plated in each of three wells in 1 ml of DMEM without phenol red supplemented with 10% fetal bovine serum, 0.01 mM non-essential amino acids, and 0.01 mM sodium pyruvate. As a negative control, three wells were supplemented with 1 ml of media without cells to observe background. Average radiance in photons/sec/cm^2^/sr was determined in the IVIS Lumina using a 10 min integration time 15 h after plating. To establish the relationship of cell number to bioluminescent flux, the average radiance values from cells producing a visible light signal under the conditions above were correlated to cell number.

### Whole animal bioluminescent imaging

Five week old nude mice were anesthetized via isoflurane inhalation until unconscious. Subjects were then subcutaneously injected with ∼5×10^6^ HEK293 cells co-transfected with pLux_CDEfrp_:CO/pLux_AB_ in a 100 µl volume of PBS. An equal number of HEK293 cells (∼5×10^6^) containing only pLux_AB_ were injected as a negative control in the same volume. The subject was imaged immediately following the injections and average radiance was determined over integration times of 1 to 10 min at intervals over a 30 min period.

### Determination of minimal detectable cell number *in vivo*

Six week old nude mice were anesthetized via isoflurane inhalation until unconscious and then injected with decreasing numbers of HEK293 cells expressing pLux_CDEfrp_:CO/pLux_AB_. In a preliminary experiment, animals were subcutaneously injected at 4 separate locations with 5 million, 2.5 million, 1 million, and 500,000 cells, each in a volume of 100 µl PBS. The subject was imaged for 10 min following injection of the final group of cells. Minimum detectable cell numbers were further delineated in a second round of injections in a fresh mouse model using cell concentrations of 500,000, 250,000, 50,000, and 25,000 cells in 100 µl PBS and identically imaged.

### Statistical analysis

Means ± S.E.M. were calculated and significant differences between groups were determined using the Student's *t*-test at *P*<0.05.

## Supporting Information

Figure S1 *luxC* codon-optimization. Alignment of the *P. luminescens* wild-type *luxC* gene (wtluxC) and the codon-optimized *luxC* gene (coluxC). Altered bases are highlighted in red.
(0.84 MB TIF)

Figure S2 *luxD* codon-optimization. Alignment of the *P. luminescens* wild-type *luxD* gene (wtluxD) and the codon-optimized *luxD* gene (coluxD). Altered bases are highlighted in red.
(4.06 MB TIF)

Figure S3 *luxE* codon-optimization. Alignment of the *P. luminescens* wild-type *luxE* gene (wtluxE) and the codon-optimized *luxE* gene (coluxE). Altered bases are highlighted in red.
(2.76 MB TIF)

Figure S4 *frp* codon-optimization. Alignment of the *V. harveyi* wild-type *frp* gene (wtfrp) and the codon-optimized *frp* gene (cofrp). Altered bases are highlighted in red.
(1.94 MB TIF)

Figure S5 pLux_CDEfrp_ in its codon-optimized and wild-type forms. Vectors were created to express the *P. luminescens luxCDE* genes responsible for aldehyde biosynthesis as well as the NAD(P)H:Flavin oxidoreductase *frp* gene from *V. harveyi* using either the (A) codon-optimized (co) or (B) wild-type (wt) gene sequences.
(1.12 MB TIF)

Figure S6 Growth curve analysis of *lux*-containing HEK293 cells. Growth curve analysis of cells containing no plasmids (negative control, untransfected HEK293) or cells containing pLux_AB_ co-transfected with either pLux_CDEfrp_:WT or pLux_CDEfrp_:CO. Cells were grown over a 96 h period until 80% confluent, representing normal passage conditions. Values are the average of three trials and are reported with the standard error of the mean.
(2.87 MB TIF)
