# Supplementary figures and images for: Autonomous Bioluminescent Expression of the Bacterial Luciferase Gene Cassette (lux) in a Mammalian Cell Line
Source: PLoS One. 2010 Aug 27;5(8):e12441. doi: 10.1371/journal.pone.0012441 (PMC2929204; doi:10.1371/journal.pone.0012441)

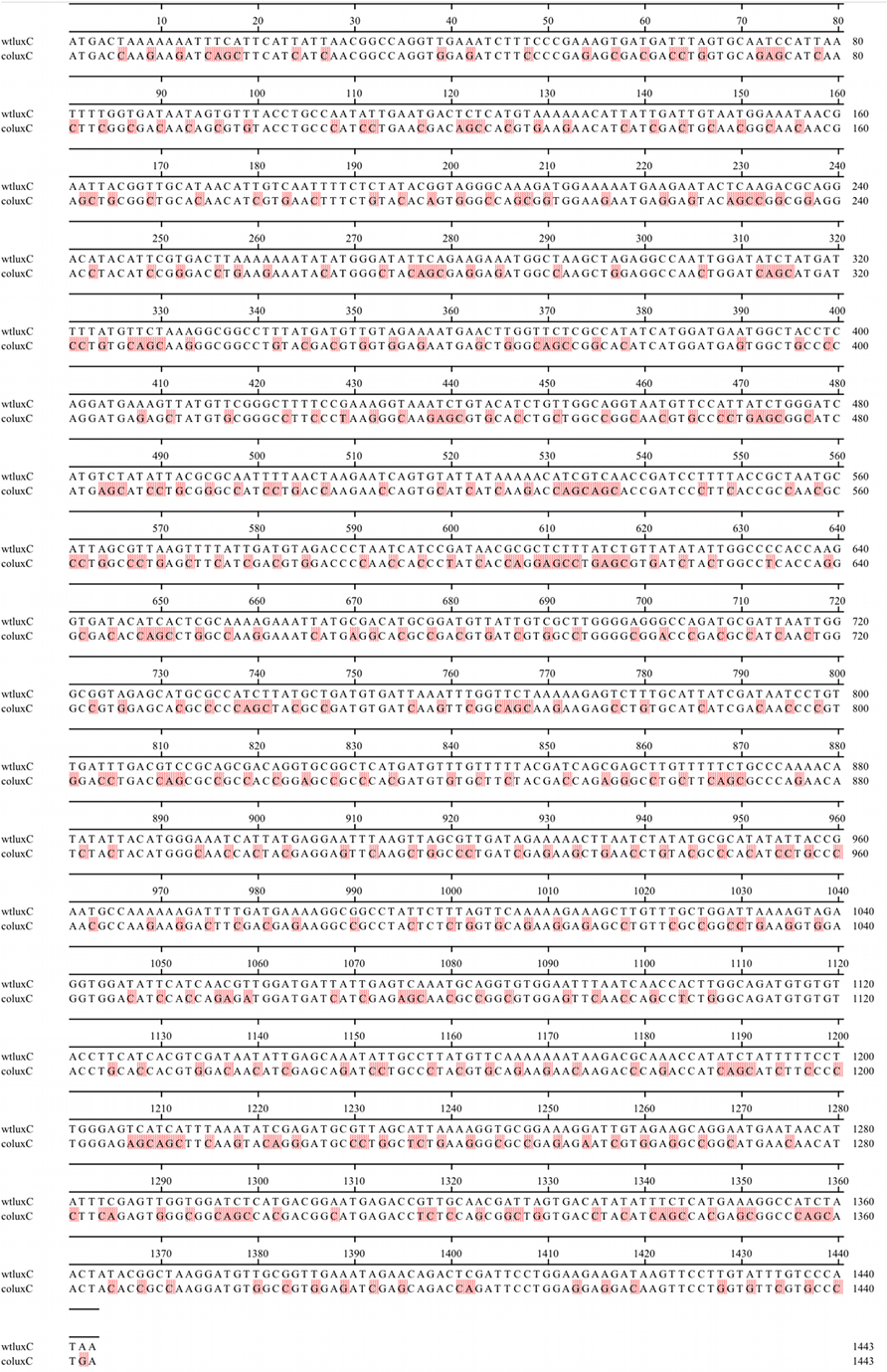

Supplement: Figure S1 — luxC codon-optimization. Alignment of the P. luminescens wild-type luxC gene (wtluxC) and the codon-optimized luxC gene (coluxC). Altered bases are highlighted in red. (0.84 MB TIF) [file pone.0012441.s001.tif]

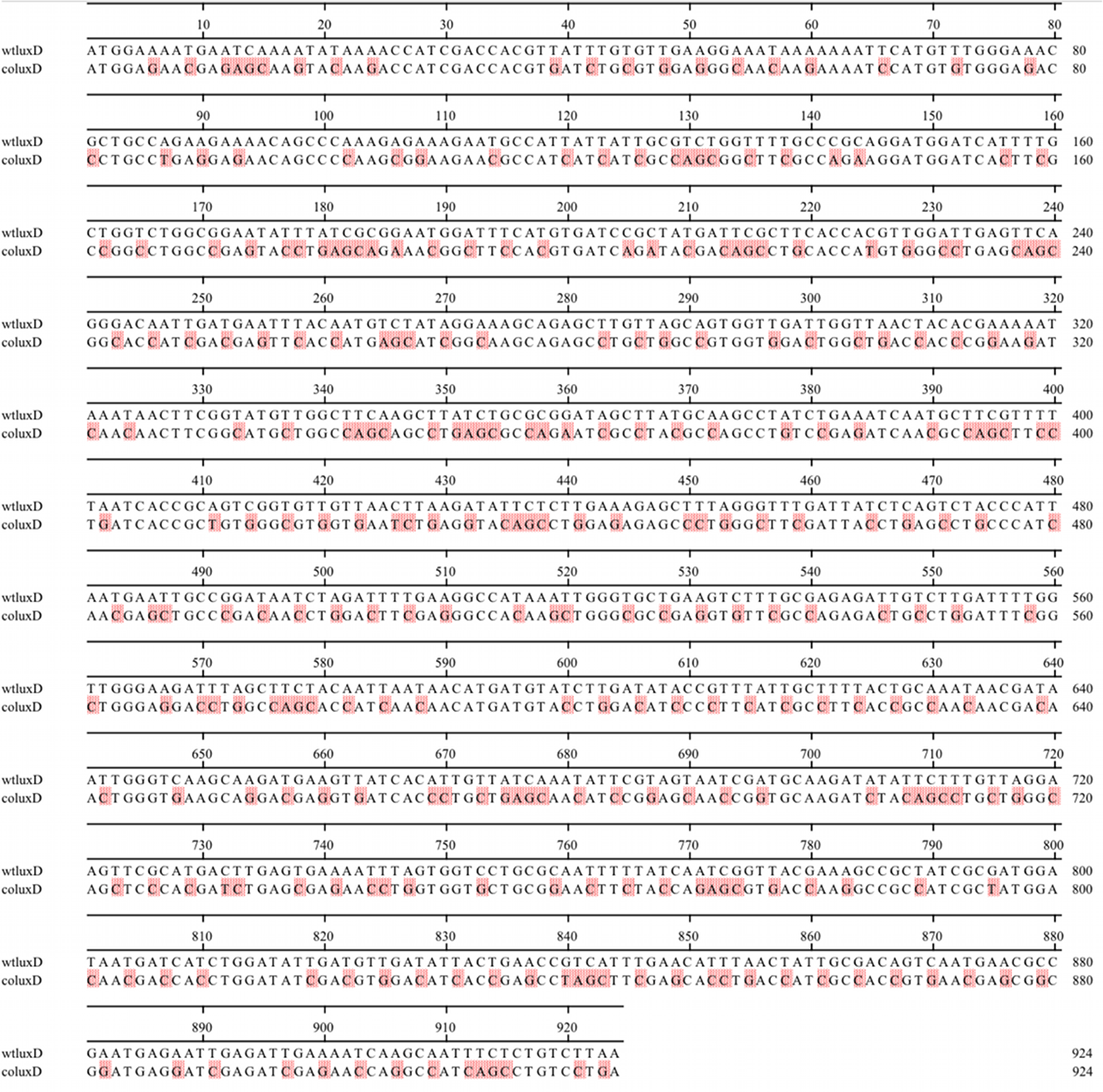

Supplement: Figure S2 — luxD codon-optimization. Alignment of the P. luminescens wild-type luxD gene (wtluxD) and the codon-optimized luxD gene (coluxD). Altered bases are highlighted in red. (4.06 MB TIF) [file pone.0012441.s002.tif]

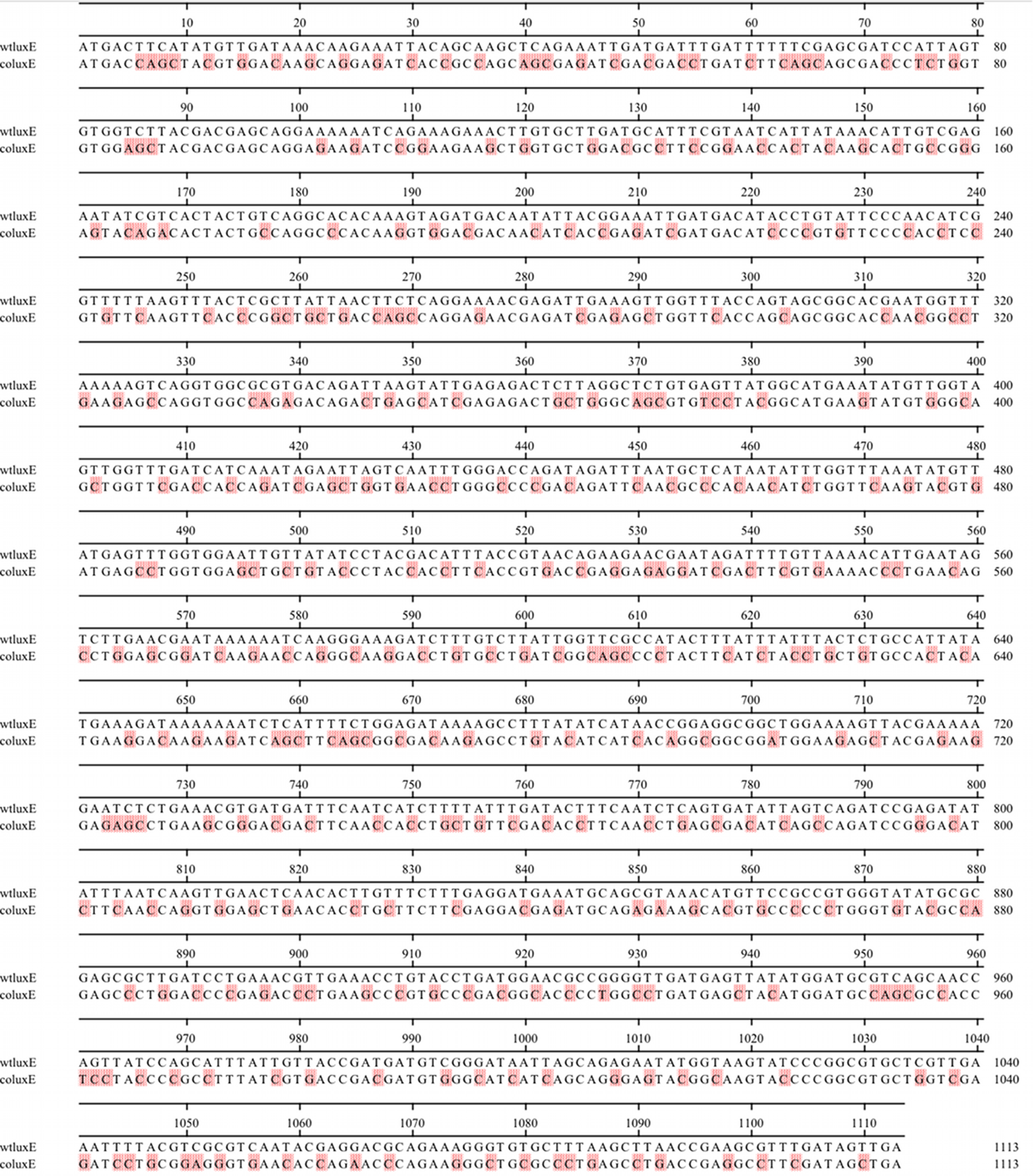

Supplement: Figure S3 — luxE codon-optimization. Alignment of the P. luminescens wild-type luxE gene (wtluxE) and the codon-optimized luxE gene (coluxE). Altered bases are highlighted in red. (2.76 MB TIF) [file pone.0012441.s003.tif]

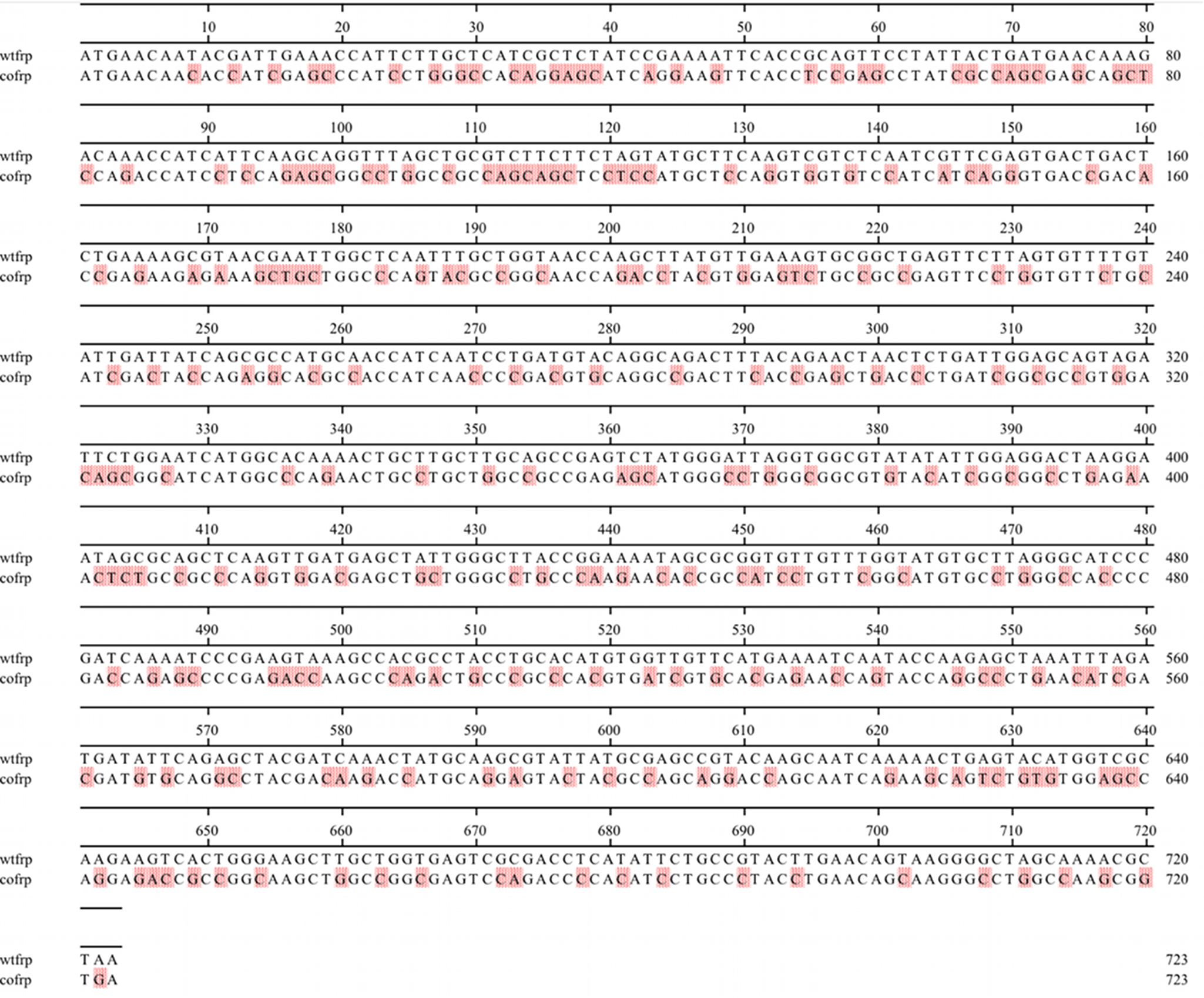

Supplement: Figure S4 — frp codon-optimization. Alignment of the V. harveyi wild-type frp gene (wtfrp) and the codon-optimized frp gene (cofrp). Altered bases are highlighted in red. (1.94 MB TIF) [file pone.0012441.s004.tif]

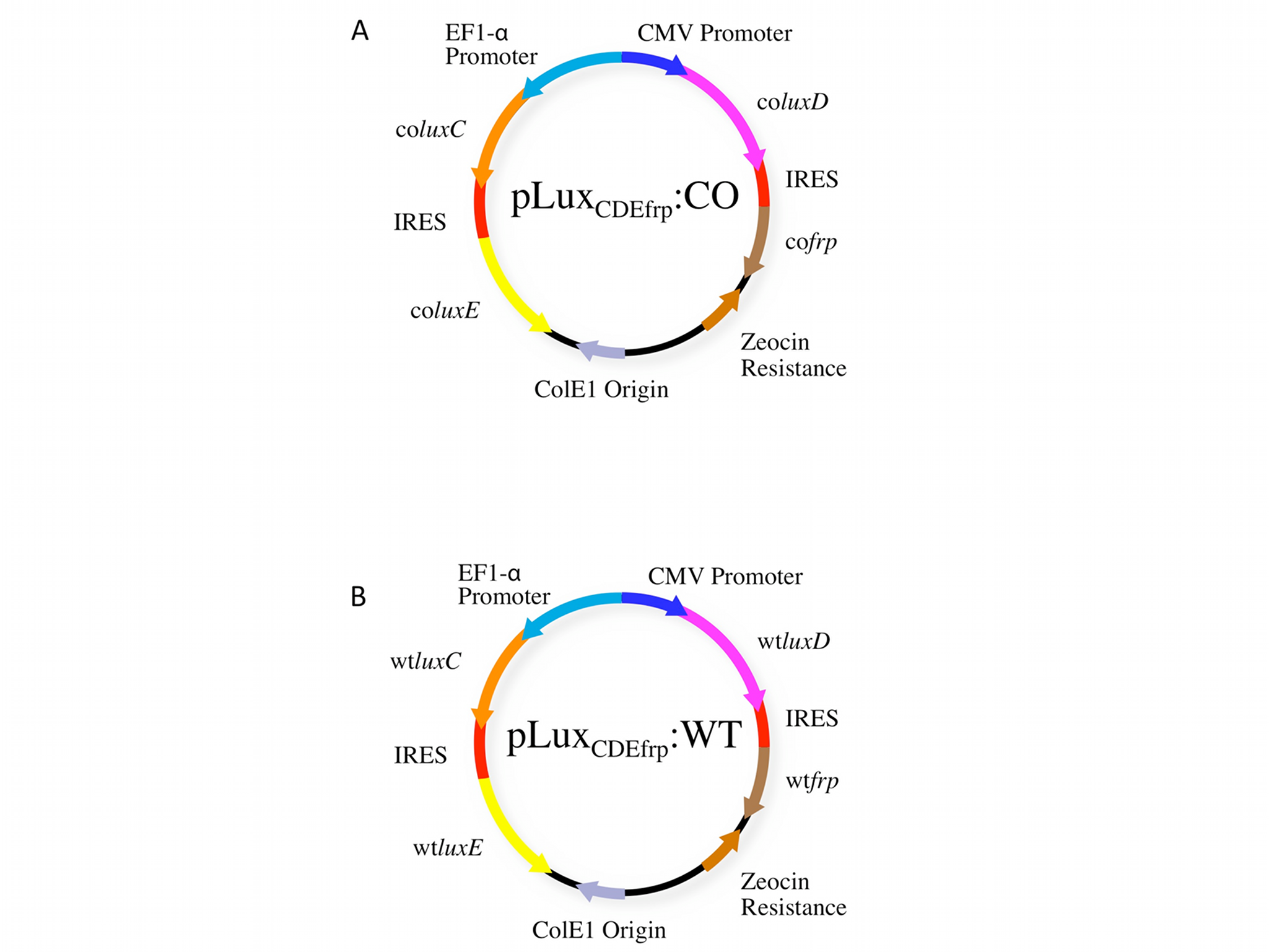

Supplement: Figure S5 — pLuxCDEfrp in its codon-optimized and wild-type forms. Vectors were created to express the P. luminescens luxCDE genes responsible for aldehyde biosynthesis as well as the NAD(P)H:Flavin oxidoreductase frp gene from V. harveyi using either the (A) codon-optimized (co) or (B) wild-type (wt) gene sequences. (1.12 MB TIF) [file pone.0012441.s005.tif]

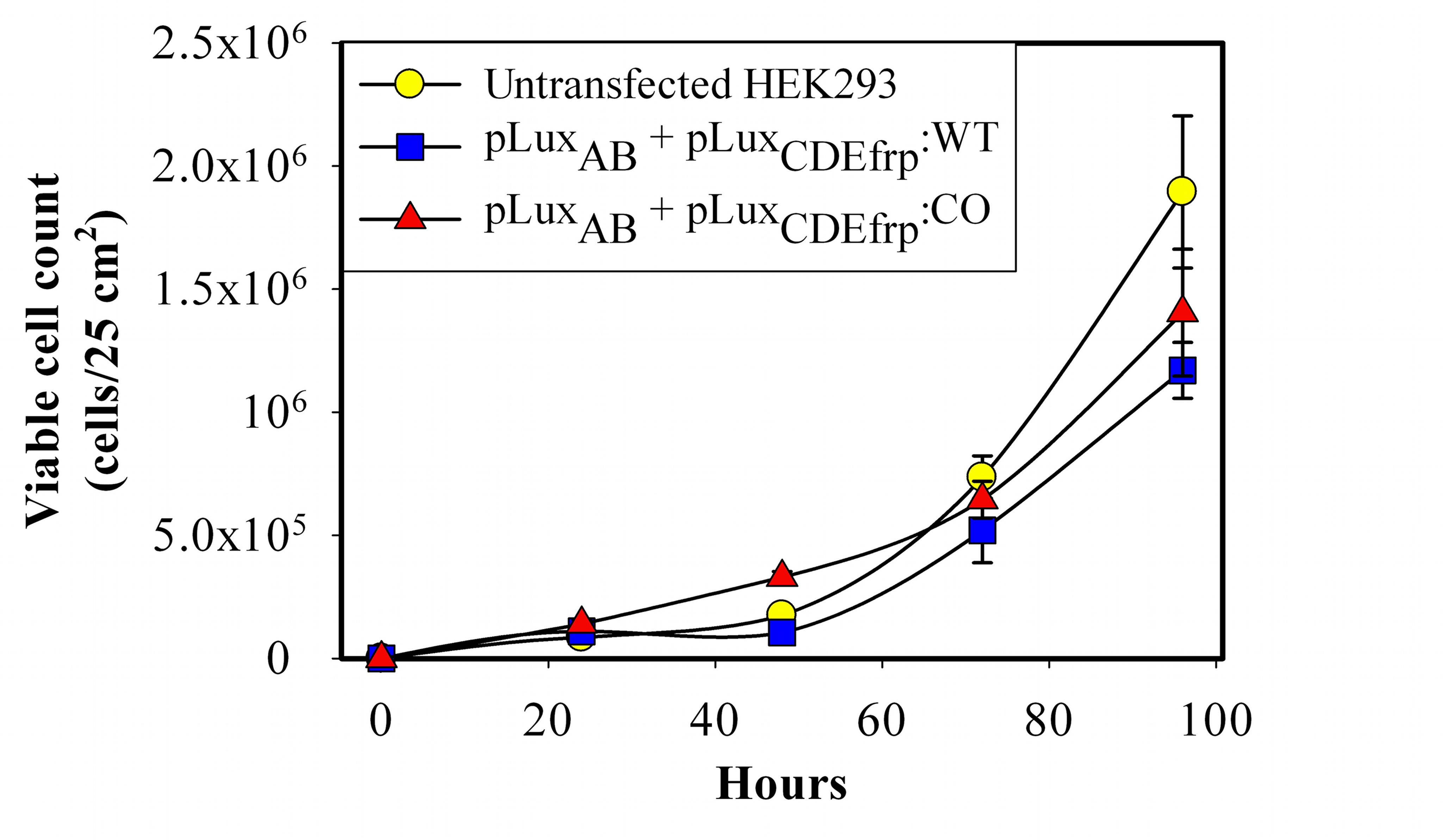

Supplement: Figure S6 — Growth curve analysis of lux-containing HEK293 cells. Growth curve analysis of cells containing no plasmids (negative control, untransfected HEK293) or cells containing pLuxAB co-transfected with either pLuxCDEfrp:WT or pLuxCDEfrp:CO. Cells were grown over a 96 h period until 80% confluent, representing normal passage conditions. Values are the average of three trials and are reported with the standard error of the mean. (2.87 MB TIF) [file pone.0012441.s006.tif]
